# Evaluation of the Success Rate of a 3D-Printed Videolaryngoscope Model in Simulation

**DOI:** 10.7759/cureus.102278

**Published:** 2026-01-25

**Authors:** Andres Duque-Estévez, German Franco-Gruntorad, Laura Peña-Blanco, Natalia Restrepo-Patiño, Juan D Gutierrez-Navarro

**Affiliations:** 1 Department of Anaesthesiology, Fundación Cardioinfantil-Instituto de Cardiología, Bogotá, COL; 2 Department of Anaesthesiology, Fundación Cardioinfantil- Instituto de Cardiología, Bogota, COL; 3 Faculty of Medicine and Health Sciences, Colegio Mayor Nuestra Señora del Rosario, Bogotá, COL

**Keywords:** airway management, intratracheal intubation, simulation in medical education, simulation training, three-dimensional (3d) printing, videolaryngoscope

## Abstract

Introduction

Orotracheal intubation (OTI) is a critical airway management procedure. Although videolaryngoscopy improves the success rates and learning curves, its high cost limits its accessibility. This study aimed to evaluate the first-attempt success rate of a low-cost, self-designed, 3D-printed videolaryngoscope in a simulation-based training scenario involving anesthesiology residents.

Methods

A simulation-based device performance assessment study was conducted to evaluate a 3D videolaryngoscope model, named Printoscope. The final design featured a blade angled at 62° and a camera positioned at 50°, both of which were optimized to enhance glottic visualization and facilitate orotracheal tube placement. The device was 3D printed and connected to a low-cost industrial borescope, a small-diameter camera system with integrated illumination. A total of 22 anesthesiology residents underwent standard manikin intubations. The variables assessed included intubation time, Cormack-Lehane grade, percentage of glottic opening (POGO), and first-attempt success rate.

Results

The first-attempt success rate was 90.91% (20/22). The mean intubation time was 28.6 seconds (range: 7.7-153 seconds). The median POGO score was 100%, and 90.9% (20/22) of the participants achieved a Cormack-Lehane Grade I view. The usability and ergonomic ratings of the device were both 5 out of 5.

Conclusions

The Printoscope is a cost-effective, reproducible, and highly usable tool for airway training simulation. Its implementation may expand access to tools for advanced airway education, particularly in resource-limited settings. Clinical validation in real patients and multicenter evaluation are planned as a pilot simulation study to further assess its applicability.

## Introduction

Orotracheal intubation (OTI) is a vital procedure for airway securing. In the United States alone, approximately 650,000 OTI procedures are performed annually outside the operating room, with 53.8% of these procedures occurring in emergency departments [1,2]. The success of this procedure largely depends on the healthcare provider’s experience and tools. Multiple attempts, prolonged laryngoscopy, and operator inexperience are associated with adverse outcomes and procedural failure [3].

According to cumulative summation (CUSUM) learning curves, approximately 50 intubation attempts are required to achieve a 90% success rate [4]. In this context, videolaryngoscopy offers a clear advantage in the training of medical students and residents, as it has been associated with a faster learning curve, fewer attempts, and shorter time to successful intubation [5-8]. However, its high cost, ranging from approximately USD 800 to 12,000, limits access to videolaryngoscopy-based training, particularly for resource-limited settings [9].

To address this gap, a low-cost videolaryngoscope named the Printoscope was designed and 3D-printed to evaluate its effectiveness in simulated airway training. This study aimed to determine the first-attempt success rate of OTI using the device among anesthesiology residents at Fundación Cardioinfantil, Institute of Cardiology, a university in Bogotá, Colombia. Additionally, it assessed variables such as time to successful intubation, glottic visualization using the Cormack-Lehane grade, and the percentage of glottic opening (POGO) score, and overall user perception of the device. The Printoscope was proposed as a cost-effective solution to improve access to advanced airway training in resource-constrained settings worldwide.

## Materials and methods

A simulation-based device performance assessment study was conducted based on the progressive development of the Printoscope, a digital, 3D model of a videolaryngoscope. The design was created using open-source software (SketchUp®; Trimble Inc., Westminster, CO, USA) and underwent multiple iterations focused on optimizing glottic visualization.

Data collection was carried out on October 18, 2024. A total of 22 anesthesiology residents (post-graduate year 1 (PGY-1) to PGY-3) from Fundación Cardioinfantil, Institute of Cardiology in Bogotá, Colombia, participated in the study. Residents were eligible if they were actively enrolled in the program, had documented prior experience with direct and video laryngoscopy as recorded in a procedural log system developed by the residency program, and agreed to participate voluntarily in the study. Residents were excluded if they were unable to attend the simulation session, had prior exposure to the 3D-printed videolaryngoscope used in this study, or were members of the research or development team. The study was approved by the Institutional Ethics Committee (approval number: 016-2024), and all participants provided written informed consent prior to inclusion.

**Figure 1:**
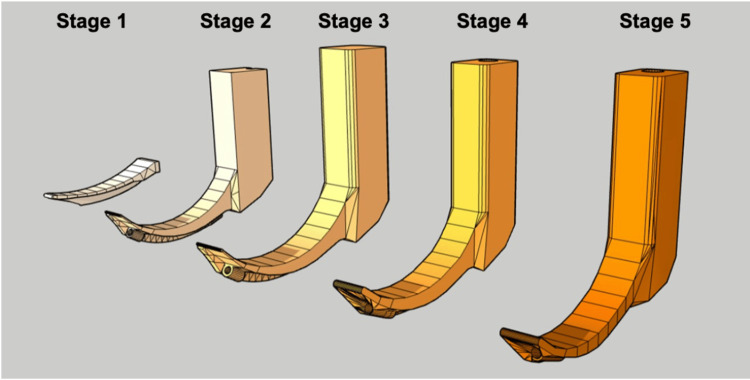
Iterative development stages of the 3D-printed Printoscope The figure illustrates the five sequential design stages that led to the final model used in this study. Stage 1: Initial digital sketch of the blade curvature; Stage 2: Integration of a basic handle structure; Stage 3: Refinement of the blade geometry and inclusion of an internal channel for the borescope; Stage 4: Optimization of blade and camera angles, ergonomics and external geometry of the handle; Stage 5: Final version prepared for 3D printing and functional testing. Image credit: Created by the authors using SketchUp®, an open-source software.

The blade was ultimately adjusted to a 62° angle to improve visualization, which was slightly greater than that of a standard Macintosh 3-blade. The camera was positioned at a 50° angle so that the vocal cords appeared in the upper third of the screen, facilitating orotracheal tube placement. Figure 1 shows a schematic representation of the Printoscope design progression.

**Figure 2:**
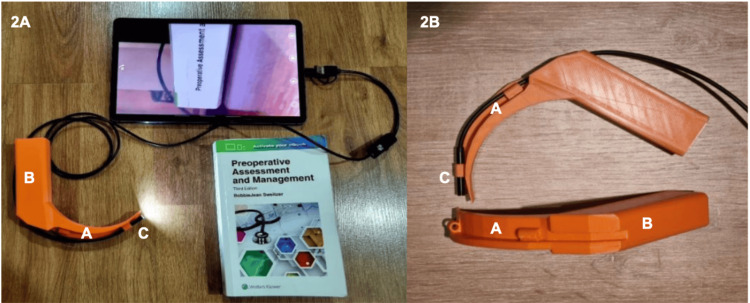
Final 3D-printed Printoscope used in this study (2A) Lateral view of the Printoscope connected to a mobile device, showing the functional imaging setup used during the simulation sessions; (2B) Close-up view highlighting the structural components of the device. The labeled elements include: (A) 3D-printed blade (62° curvature), (B) ergonomic handle with finger groove and smartphone attachment point, and (C) integrated industrial borescope (IP67) with LED illumination positioned at a 50° angle. The system provides a focal range of 3–8 cm and enables real-time visualization through any compatible smartphone or tablet. Image credit: Authors

The device was designed to be compatible with a low-cost industrial borescope, which is water-resistant (IP67), equipped with integrated lighting, and has a focal range of 3-8 cm. This borescope connects to any smartphone or tablet and provides real-time laryngoscopy imaging. The entire Printoscope structure was 3D-printed using polylactic acid (PLA), and the borescope is commercially available, with a total manufacturing cost of approximately 16 USD per unit. The final design was tested in a clinical simulation setting to validate its educational utility for airway management training (Figure 2).

**Figure 3:**
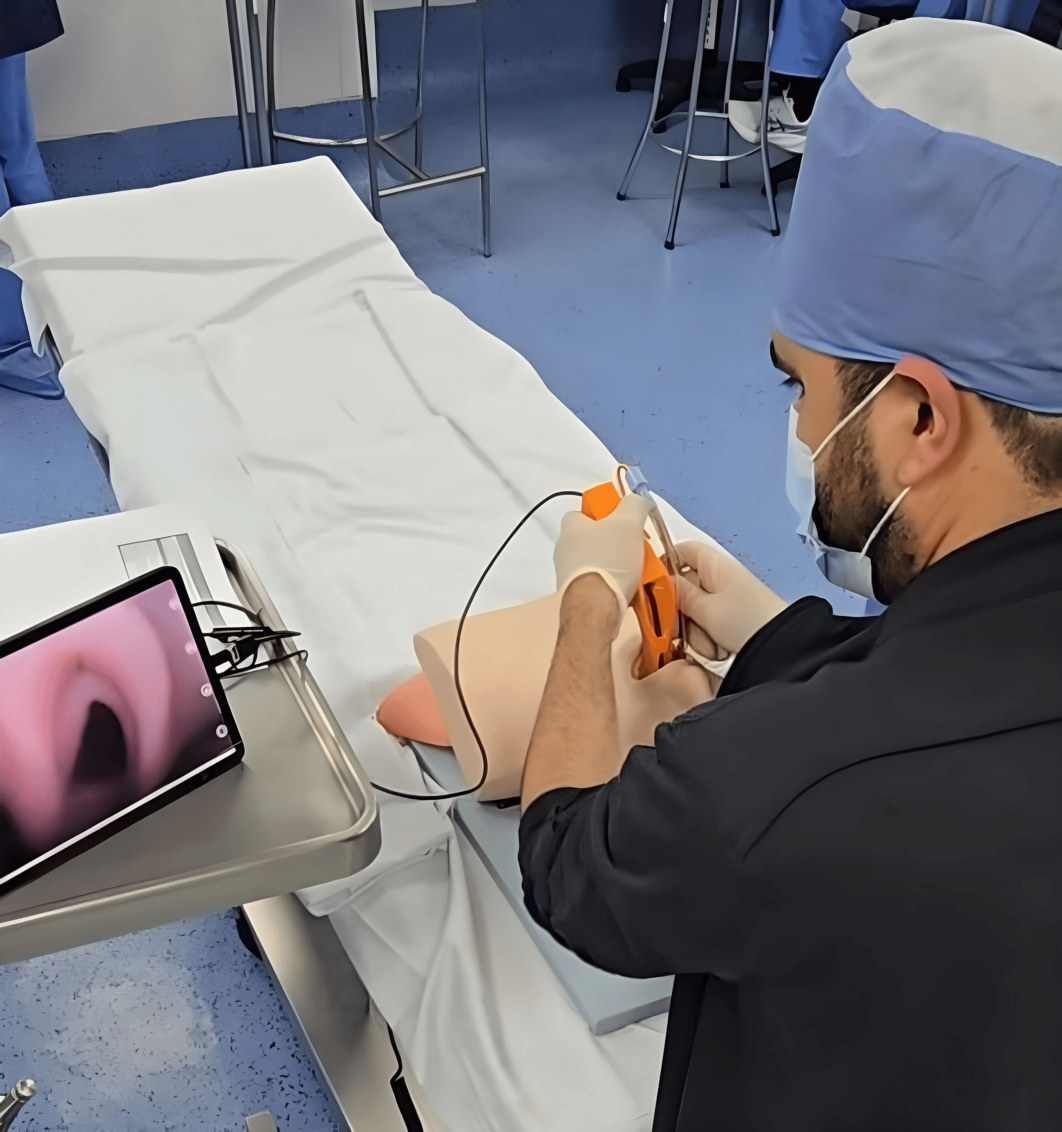
Anesthesiology resident performing orotracheal intubation on a manikin with the Printoscope. Image credits: Authors; the resident gave written permission to use and distribute the image.

Simulations were conducted in a standardized surgical simulation room at a high-complexity institution in Bogotá, Colombia. All residents used the same Printoscope model and manikin under controlled rapid-sequence intubation conditions (Figure 3). The only individualized variable was surgical table height, which was adjusted according to each participant’s preference.

Before each simulation, participants were instructed on the step-by-step process to achieve both the primary and secondary objectives. Timing began when the laryngoscope was grasped and ended when endotracheal cuff inflation was indicated.

After the simulation session, each participant completed an anonymous subjective evaluation form and documented the glottic view using the Cormack-Lehane classification, which ranges from Grade I (full glottic visualization) to Grade IV (no identifiable glottic structures) [10]. Glottic exposure was also quantified using the POGO score, calculated as the proportion (0%-100%) of the glottis visible from the anterior to the posterior commissure, where 0% indicates no visualization and 100% denotes complete visualization [11].

Each participant completed a single simulation session with one intubation attempt to avoid learning effects from repeated exposure. The procedure was deemed successful when the lead investigator confirmed correct tracheal tube placement via direct visualization through the manikin. If the orotracheal tube was found in the esophagus, the attempt was classified as failed, and a new attempt was performed under identical conditions until successful intubation was achieved. Subsequent attempts were excluded from the primary analysis, as the objective was to assess first-attempt success. The procedure followed a standardized and replicable sequential flow. 

Statistical analysis

Descriptive statistics were used to summarize the data. Continuous variables are reported as mean (SD) or median (IQR), depending on distribution, and categorical variables as n (%). No inferential statistical comparisons were performed, given the exploratory and pilot nature of this study.

## Results

All 22 first- to third-year anesthesiology residents met the inclusion and exclusion criteria. To ensure consistency of analysis, only the first intubation attempt of each resident was considered. Participants reported a median of 28.59 prior videolaryngoscopes (range: 0-80) and 289 direct laryngoscopy (range: 22-850), with experience increasing progressively by residency year. Table 1 presents the demographic characteristics and prior airway management experience of the patients.

**Table 1:** Demographic characteristics of the study group Continuous variables are presented as mean (standard deviation) or median (interquartile range) as appropriate. Categorical variables are presented as n (%).

Variable	n= 22
Age (years)	29.3 (2.6)
Residency year	
First year	7 (31.8%)
Second year	8 (36.4%)
Third year	7 (31.8%)
Sex	
Female	11 (50%)
Male	11 (50%)
Previous experience	
Number of direct laryngoscopies	344.4 (256.4)
Number of videolaryngoscopies	28.6 (23.1)

The overall first-attempt success rate was 90.91%, with two residents failing to successfully intubate. Table 2 summarizes the general results and comparisons by experience level. The average intubation time was 28.60 seconds (range: 7.70-153 seconds), which notably decreased with higher experience and training level, with the fastest times achieved by third-year residents.

**Table 2:** Variable distribution by residency year and device evaluation Continuous variables are presented as median (IQR) or mean (SD) as indicated. Categorical variables are presented as n (%). Success on first attempt: number of participants with successful first attempt/total in that residency year (percentage). Cormack–Lehane grades are presented in separate rows as Grade I, Grade II, and Grade IV (%) (No Grade III was reported). Overall rating, Visualization rating, Ergonomics rating: ordinal scales from 1 (worst) to 5 (best). POGO: percentage of glottic opening; PGY: post-graduate year

Variable	PGY-1 (n=7)	PGY-2 (n=8)	PGY-3 (n=7)	Total (n=22)
Previous videolaryngoscopies	3 (1-10)	30.5 (24.75-33.5)	60 (27-60)	–
Previous direct laryngoscopies	64 (27-131)	289 (231.5-386)	599 (508-838)	–
Intubation time (sec)	25.2 (19.6-34.8)	19.15 (16.63-25.95)	19 (15.1-36.2)	20.45 (17,8-29,9)
Success on first attempt (%)	6 (85.7%)	7 (87.5%)	7 (100%)	20 (90.91%)
POGO (%)	100 (90-100)	100 (85-100)	100 (100-100)	100 (100-100)
Cormack I (%)	6 (85.7%)	7 (87.5%)	7 (100%)	90.91%
Cormack II (%)	1 (14.3%)	0	0	4.55%
Cormack IV (%)	0	1 (12.5%)	0	4.55%
Overall rating	4 (4-5)	5 (4-5)	4 (4-5)	5 (4.25–5)
Visualization rating	5 (5-5)	5 (4-5)	4.7 (4-5)	5 (5–5)
Ergonomics rating	5 (4-5)	5 (4-5)	5 (4-5)	5 (5–5)

Regarding visualization, the median POGO score was 100%. A total of 90.9% (20 of 22) of patients reported a Cormack-Lehane Grade I view. One of the two failed intubations was associated with a grade IV view. The device received a usability and ergonomic rating of 5 out of 5, reflecting a highly positive perception among the participants.

Finally, the participants were divided based on their CUSUM learning curve data for videolaryngoscopy using a threshold of 20 prior procedures. The “expert” subgroup (≥20 videolaryngoscopies) completed successful intubation an average of 14.8 seconds faster than their less experienced peers. Both subgroups had one failed intubation attempt. These findings suggest that the Printoscope enables efficient performance even among operators with limited prior experience.

## Discussion

OTI is a fundamental procedure in airway management in which appropriate training and operator skills directly influence success rates. In this study, the Printoscope, a 3D-printed videolaryngoscope, achieved a first-attempt success rate of 90.91%, closely approaching the performance reported by Asan et al. [6] with commercial videolaryngoscopies in simulation-based studies, where first-pass success rates of 95% to 100% were observed. This difference may be attributed to the dissimilar blade or camera angulations or the manikin used for the simulations; however, direct comparisons are limited by differences in study protocols. This study’s findings are consistent with the emerging role of 3D printing in airway management and medical education [12], which has demonstrated feasibility across different devices and training contexts.

The Printoscope also outperformed other 3D-printed models, such as the AirAngel, which reported a success rate of only 47.80% [13]. The hyper-angulated blade of AirAngel (>70°) is likely to explain this significant difference, which may induce Kovacs’ sign [14], hindering tracheal alignment and complicating intubation. In contrast, the moderate blade angle of the Printoscope (62°) appears to optimize laryngoscopy, while the 50° angulation of the camera optimizes visualization and tube insertion.

A key advantage of the Printoscope is its excellent airway visualization, as demonstrated by a median POGO score of 100%, which exceeds the 50%-80% range typically reported with commercial devices [15]. This outcome was achieved through a design tailored to the optimal focal length of the borescope (3-8 cm) and a 50° camera angle that positioned the vocal cords in the upper third of the screen, facilitating intubation maneuvers.

Another notable advantage of the device is its affordability: the Printoscope offers a scalable solution, particularly relevant in resource-limited settings, at USD 16 per unit compared to USD 800-12,000 for commercial videolaryngoscopies. This low cost could democratize videolaryngoscopy training and allow its widespread implementation in simulation centers.

Beyond affordability, the Printoscope may address some structural barriers to airway management training in low-resource settings. In many training programs, access to commercial videolaryngoscopies is limited by cost, availability, and concerns regarding equipment damage, which can restrict hands-on practice for trainees. A low-cost, reproducible 3D-printed device allows repeated use in simulation-based curricula without these constraints, facilitating early exposure to videolaryngoscopy principles for residents and medical students. This approach may support more equitable access to airway training, particularly in institutions with limited simulation resources or constrained educational budgets.

This study has some limitations. First, although all participants were anesthesiology residents, their prior experience varied; 36% had performed fewer than 20 videolaryngoscopies and thus did not meet the threshold to be classified as “experienced.” Nonetheless, OTI times and success rates were similar between the subgroups. As a pilot, simulation-based evaluation without a comparator group, the sample size was intentionally limited and reflects the exploratory educational focus of the study. Before minimizing bias, the participants were not allowed to handle or observe the device to ensure standardized exposure and avoid performance bias. Additionally, the subjective evaluation instrument used in this study was not formally validated, which may limit the interpretability of resident-reported perceptions. 

Selection bias was addressed by including all residents who met the inclusion and exclusion criteria. Measurement and observer bias were also mitigated by using the same digital timer for all participants and ensuring the presence of two evaluators throughout the simulation sessions. Given its exploratory nature, this study should be considered a pilot evaluation that provides preliminary evidence to guide future multicenter and clinical validation studies.

Finally, this study was conducted exclusively on manikins; extrapolation to real clinical settings is limited. Additionally, the participants were exclusively anesthesiology residents, whose experience may exceed that of learners from other medical specialties. Further studies are warranted to evaluate the device’s performance in actual patient care and across diverse clinical disciplines.

## Conclusions

The Printoscope, a low-cost 3D-printed videolaryngoscope, was demonstrated to be an effective device for OTI training in simulation, with a high first-attempt success rate. These findings support its potential use as an accessible training tool for residents and medical students in simulated learning environments, particularly in resource-limited settings, particularly in settings where access to commercial training devices may be limited. Further studies are needed to evaluate its educational impact across diverse learner populations and training contexts. 
